# The relationship between kidney function and the soluble (pro)renin receptor in young adults: the African-PREDICT study

**DOI:** 10.1186/s12882-025-04038-x

**Published:** 2025-04-03

**Authors:** Phuti J. Mokgonyana, Gontse G. Mokwatsi, Stella M. Gwini, Lebo F. Gafane-Matemane

**Affiliations:** 1 Hypertension in Africa Research Team (HART), North-West University, Potchefstroom Campus, Private Bag X6001, Potchefstroom, 2520 South Africa; 2 MRC Research Unit for Hypertension and Cardiovascular Disease, North-West University, Potchefstroom Campus, Private Bag X6001, Potchefstroom, 2520 South Africa; 3 School of Public Health and Preventive Medicine, Monash University, Melbourne, Australia

**Keywords:** Kidney dysfunction, Estimated glomerular filtration rate, (Pro)renin receptor, Renin angiotensin-aldosterone system, Soluble (pro)renin receptor

## Abstract

High renin angiotensin-aldosterone system (RAAS) activity is associated with target organ damage. Soluble (pro)renin receptor [s(P)RR] forms part of the RAAS cascade and is associated with kidney damage through both angiotensin II-dependent and -independent pathways. Additionally, s(P)RR levels are higher in hypertension and chronic kidney disease (CKD) patients. However, little is known regarding ethnic and sex differences in s(P)RR levels and its potential associations with kidney function in young healthy adults. Identifying these associations in young populations is essential for identification of areas of intervention to prevent CKD. This study aimed to compare levels of s(P)RR across ethnic and sex groups and determine s(P)RR associations with markers of kidney function, including estimated glomerular filtration rate (eGFR), urine albumin-to-creatinine ratio (uACR) and alpha 1-microglobulin (uA1M). The study included 1156 young healthy Black and White South Africans aged 20–30 years (Men, *N* = 555; Women, *N* = 601). We measured uA1M, albumin and creatinine in urine to calculate uACR. s(P)RR, cystatin C and creatinine were measured in serum and eGFR was calculated. Independent t-tests and multiple regression analyses were carried out to compare groups and explore associations. s(P)RR levels were higher in White participants, and higher in White men than in women (all *p* < 0.001). eGFR was higher in both Black men and women than in White men and women (both *p* ≤ 0.001). Both uA1M and uACR were higher in Black men than in White men (both *p* ≤ 0.003). We observed an independent negative association between eGFR and s(P)RR in Black women only (Adj.R^2^ = 0.309; Std. β=-0.141; *p* = 0.026), while uA1M associated positively with s(P)RR in the White group only (Adj.R^2^ = 0.063; Std. β = 0.115; *p* = 0.018). No associations were evident between uACR and s(P)RR in any of the groups. The positive association between uA1M and s(P)RR suggest that s(P)RR may contribute to kidney damage in young White participants through pathways associated with inflammation and fibrosis. A better understanding of mechanisms linking s(P)RR to kidney damage may lead to discovery of areas of therapeutic interventions for the prevention and treatment of CKD in different population groups.

**Trial registration** ClinicalTrials.gov NCT03292094. Registration date 2017-09-12.

**Supplementary Information:**

The online version contains supplementary material available at 10.1186/s12882-025-04038-x.

## Introduction

The renin angiotensin-aldosterone system (RAAS) is essential for fluid and electrolyte balance maintenance [1, 2]. Direct effects of RAAS are mostly exerted via the actions of angiotensin II (Ang II) and the (pro)renin receptor [(P)RR], one of the components of the RAAS cascade discovered over the past two and a half decades [3–5]. Tissue (P)RR can exert deleterious renal and cardiovascular effects via Ang II-dependent and independent pathways as indicated in animal studies, with data in humans being scarce [6–10]. The cleavage of (P)RR by furin yields the soluble form of (P)RR, known as the soluble (pro)renin receptor [s(P)RR] [11]. Similar to (P)RR, s(P)RR activation also mediates angiotensinogen cleavage, binds and activates prorenin/renin, and contributes to its RAAS-dependent and independent effects [12].

Xie et al. [13] found that s(P)RR upregulates fibronectin in human kidney proximal tubular cells via the phosphatidylinositol 3-kinase/Akt/β-catenin/snail signalling pathway. (P)RR and s(P)RR contributes to kidney inflammation and fibrosis in a gradual process via activation of the wingless Type β-catenin and mitogen-activated protein kinase 1/2 signalling pathway, enhancing the expression of downstream profibrotic markers such as fibronectin and plasminogen activator inhibitor-1 (PAI-1), and the activation of inflammatory and fibrotic molecules including tumour necrosis factor-α, transforming growth factor-β, PAI-1 and interleukin-6 (IL-6) [14, 15]. Elevated s(P)RR levels have been found in the elderly and individuals with underlying conditions such as primary aldosteronism [16], kidney dysfunction [17, 18], and hypertension in human and animal studies [11].

Furthermore, s(P)RR has been linked to the progression of kidney disease in several clinical studies [18, 19] that included chronic kidney disease (CKD) patients, diabetic and non-diabetic men and women. This highlights the need for studies that focus on healthy and young individuals to provide evidence for early prevention. Compared to other RAAS components, it is not known whether the potential associations of s(P)RR with kidney function may be influenced by sex and ethnicity. Lower levels of s(P)RR were found in young Black men compared to White men [20], an observation that was recently confirmed in a sub-study of the current population [21], while men had higher s(P)RR concentrations than women [22].

Plasma s(P)RR levels are positively associated with urinary protein levels [18, 23] and negatively with estimated glomerular filtration rate (eGFR) [17, 24], as indicators of kidney function [25]. Recent studies have shown that alpha 1-microglobulin (A1M), a multifunctional immunomodulatory protein [26], may be a more sensitive marker of kidney function [27] compared to conventional markers including serum creatinine, urea levels and cystatin C [28]. A1M is positively associated with urine albumin-creatinine ratio (uACR) and negatively correlated with eGFR [29, 30]. This protein is found in blood in its unbound form and in complexes with albumin and immunoglobulin A [26]. The unbound form is freely filtered by the kidney due to its low molecular weight [31] and is then reabsorbed by the proximal tubular cells [32]. The main function of A1M is thought to be protective against oxidative stress and promote the body’s inherent tissue healing mechanisms [33]. In circumstances of reduced eGFR, both serum and urinary A1M (uA1M) are elevated due to kidney tubular abnormalities [34, 35].

To the best of our knowledge there are no data on the association between uA1M and plasma s(P)RR concentrations in young adults. Even though the association between eGFR, proteinuria and serum s(P)RR has been explored in older and diseased populations, further investigations need to focus on healthy populations, while considering sex and ethnic differences. This young population represents a phase where kidney function is typically optimal and RAAS activity well-regulated without confounding effects of age-related kidney function decline or comorbidities such as diabetes and hypertension. Therefore, we explored sex and ethnic differences in s(P)RR levels and investigated the associations between markers of kidney function (eGFR, uACR and uA1M) and s(P)RR in young, apparently healthy Black and White South Africans.

## Methods

### Study design and participants

This study forms part of the larger African Prospective study on Early Detection and Identification of Cardiovascular disease and Hypertension (African-PREDICT) and used existing baseline data that were collected from 2013 to 2017. The African-PREDICT study has a longitudinal design, aimed at identifying novel and early markers of cardiovascular risk and investigating early cardiovascular disease-related pathophysiology [31]. Cross-sectional baseline data used in the present sub-study included a population sample of 1202 young, healthy, Black and White individuals aged 20–30 years. This young population represents a phase where kidney function is optimal without confounding effects such as age-related decline in kidney function. The inclusion criteria included brachial office blood pressure (BP) of < 140 and < 90 mmHg; human immunodeficiency virus (HIV) uninfected; ability to read or understand English; no previous diagnosis or on medication for chronic disease. Pregnant or breastfeeding women were excluded. A detailed inclusion and exclusion criteria for the larger study was previously published in the protocol [31]. In addition, this sub-study excluded participants with missing data for main dependent (eGFR, uACR and uA1M) and independent (s(P)RR) variables (*N* = 46) resulting in a total of *N* = 1156 participants. All participants gave verbal and written informed consent. The African-PREDICT study was approved by the Health Research Ethics Committee (HREC) of the North-West University (NWU-00001-12-A1) and is registered on ClinicalTrials.gov (NCT03292094). All procedures followed the Declaration of Helsinki.

### Questionnaires

A general health and demographic questionnaire was completed by participants to obtain information on age, sex, ethnicity, self-reported alcohol and tobacco use, medication usage and medical history. The socioeconomic status (SES) of participants was calculated using a point system adapted from Kuppuswamy’s socio-economic status scale 2010 [36] for a South African environment, scoring participants in three categories: skill level, education and household income. Scores were used to categorise according to low, middle and high socioeconomic groups [31].

### Anthropometric and physical activity measurements

Anthropometric measurements included body height (m) (SECA 213 Portable Stadiometer 15, Hamburg, Germany), waist circumference (WC) (cm) (Lufkin Steel Anthropometric Tape W606 PM; Lufkin, Apex, USA) and weight (kg) (SECA 813 Electronic Scales, Hamburg, Germany). All measurements were performed according to guidelines described by the International Society for the Advancement of Kinanthropometry (ISAK) [37]. The body mass index (BMI) [weight (kg) / height (m^2^)] was determined. Participants were also fitted with a compact, chest-worn accelerometric device (ActiHeart 4 CamNtech Ltd., UK) which was used to quantitatively measure physical activity for the calculation of total energy expenditure (TEE) (indexed by weight and expressed as kCal//day). The ActiHeart device was worn for a maximum of 7 days.

### Cardiovascular measurements

A validated 24-hour ambulatory BP monitor (ABPM) and ECG apparatus (CardioXplore, MediTech, Budapest, Hungary British Hypertension Society) were used to collect 24-hour BP measurements. The ABPM apparatus was programmed to take BP recordings every 30 min during the day (06:00 to 22:00) and every hour during the night (22:00 to 06:00). An appropriately sized cuff was fitted to the participants’ non-dominant arm with instructions to ensure successful inflations over a 24-hour period. We achieved 88% successful mean inflation rate (standard deviation ± 12.2) over the 24-hour period that ambulatory devices were worn by participants.

### Biological sampling and biochemical analyses

Early morning spot urine samples and fasting blood samples were collected by a registered nurse. Participants were required to fast from 22:00 the evening prior to the measurement day. The blood and urine samples were immediately taken to the on-site temperature-controlled laboratory and aliquoted into cryovials for short- and long-term storage in bio-freezers at -80 °C.

Serum samples were analysed for the lipid profile (total cholesterol (TC), high density lipoprotein cholesterol (HDL-C), low density lipoprotein cholesterol (LDL-C) and triglycerides), C-reactive protein (CRP), gamma-glutamyltransferase (GGT), creatinine, cystatin C whole blood for glycated haemoglobin (HbA1c) and plasma for glucose, all using the Cobas Integra^®^ 400 plus (Roche, Basel, Switzerland). Serum cotinine was analysed using a chemiluminescence method on the Immulite 1000 (Siemens, Erlangen, Germany). A s(P)RR Assay Kit -Immuno-Biological Laboratories Co., Ltd (IBL-Japan) was used for serum s(P)RR [38, 39], with an intra assay variability (%CV) of 1.93 and inter assay variability (%CV) of 2.00. Currently, s(P)RR measurements are conducted using enzyme-linked immunosorbent assay (ELISA) kits to assess in body fluids [40] including the blood [41] and urine [42], providing an opportunity to investigate the regulation of (P)RR synthesis, s(P)RR secretion and associations with regulatory functions of RAAS [25]. Creatinine and albumin were determined using spot urine samples obtained early in the morning when participants arrived at the research facility and their ratio (uACR) was calculated. Additionally, spot urine sample was used to measure uA1M as part of a bead-based multiplex immunoassay, which allows the simultaneous quantification of multiple markers. This immunoassay was performed on a Luminex 200 system (Luminex, Austin, Texas, USA). Moreover, the serum levels of creatinine and cystatin C were used to calculate eGFR. The eGFR calculation used the Chronic Kidney Disease Epidemiology (CKD-EPI) Formula (without race factor) [43, 44].

Electrolytes, including 24-hour urinary sodium (Na^+^) and potassium (K^+^), were measured by means of ion-selective electrode potentiometry on the Cobas Integra^®^ 400 plus (Roche, Basel, Switzerland); the results were then used to calculate the 24-hour urinary sodium: potassium ratio (Na^+^: K^+^). A 24-hour urine sample was collected by each participant on a day convenient for them. Participants were asked to collect all urine passed after the first urination of the day and the first urine of the next day, and to discard both first urines. It was noted when the urine collection process began and ended. Urine samples were aliquoted and stored in a − 80 °C freezer until analyses.

### Statistical analyses

Data analyses were performed with IBM^®^ SPSS^®^ Statistics version 29 software (IBM Corporation, Armonk, New York, USA) and figures were drawn with GraphPad PRISM version 5.03. All variables were tested for normality using visual inspection (QQ-plots) and skewness and kurtosis coefficients. If skewed (non-Gaussian distributions), data were logarithmically transformed. Normally distributed data were presented as mean and standard deviation, and logarithmically transformed variables were presented by the geometric mean with 5th and 95th percentile intervals. Categorical data were presented as frequency counts, proportions and percentages. We divided the groups according to ethnicity and sex based on the previously reported ethnic differences in RAAS activity, (including s(P)RR in a sub-study including participants of the African-PREDICT study) and the well-known sex differences in kidney function [20–22, 45]. Comparisons were performed by making use of independent T-tests for continuous variables and chi-square analyses for categorical data. Analyses of covariance (ANCOVA) were used to perform appropriate interaction tests for ethnicity or sex interactions on the association between the dependent (markers of kidney function namely, eGFR, uACR, and A1M) and independent [s(P)RR] variables. Single, partial, and multiple regression analyses using the enter method were performed to determine associations between markers of kidney function (eGFR, uACR, uA1M) and s(P)RR levels. Based on exploratory Pearson correlations, the following variables were selected for inclusion in the multivariable regression analysis as confounders due to their link with markers of kidney function and components of the RAAS: triglycerides, CRP, GGT, glucose, cotinine, SES score, 24-hour systolic blood pressure (SBP), TEE, 24-hour urinary Na^+^: K^+^, WC and age. A p-value of <0.05 was regarded as statistically significant.

## Results

The characteristics of the study population stratified according to sex and ethnicity are shown in Table 1. Black participants constituted a larger proportion of the low SES class, and the number of White participants was higher in the medium and high SES classes (all *p* ≤ 0.001). In men, 24-hour SBP was higher in the White group than in their Black counterparts (*p* < 0.001). Twenty four-hour heart rate was higher in Black women than in White women (*p* < 0.001). GGT and HbA1c were both higher in Black men and women (all *p* < 0.001), whereas CRP levels were higher in Black women than in White women (*p* < 0.001). When comparing lipids, TC, triglycerides and LDL-C were higher in both White men and women than in the Black groups, while HDL-C was higher in White women and Black men (all *p* ≤ 0.012) than in Black women and White men, respectively. Higher measures of adiposity (BMI and WC) were observed in Black women than in White women and also higher in White men than in Black men (all *p* ≤ 0.017). Regarding kidney function, eGFR was higher in both Black men and women than in White men and women (both *p* ≤ 0.001). Both uA1M and uACR were higher in Black men than in White men (both *p* ≤ 0.003). s(P)RR was higher in both White men and women (both *p* < 0.001) than in their Black counterparts. When comparing electrolytes, higher 24-hour urinary Na^+^ and Na^+^: K^+^ ratio levels were detected in both Black men and women (all *p* < 0.001), while 24-hour urinary K^+^ was higher in both White men and women (both *p* < 0.001). Self-reported smoking was higher in White women and Black men than in Black women and White men (both *p* ≤ 0.005). Lastly, TEE was higher in White men compared to Black men (*p* > 0.001).

**Table 1:** The comparison of the characteristics of the study participants stratified according to sex and ethnicity

	Women			Men		
	*N* = 601			*N* = 555		
	Black	White	p-value	Black	White	p-value
	*N* = 296	*N* = 305		*N* = 278	*N* = 277	
**Socio-demographic profile**						
Age (years)	24.5 ± 3.28	24.4 ± 3.05	0.63	24.4 ± 3.06	24.7 ± 3.03	0.17
SES class			**< 0.001**			**< 0.001**
Low n/total (%) Medium n/total (%) High n/total (%)	161/296 (54.4%)	62/305 (20.3%)		176/277 (63.5%)	52/277 (18.8%)	
	90/296 (30.4%)	95/305 (31.1%)		67/277 (24.2%)	87/277 (31.4%)	
	45/296 (15.2%)	148/305 (48.5%)		34/277 (12.3%)	138/277(49.8%)	
**Blood pressure measurements**						
24-hour SBP (mmHg)	112 ± 8.47	112 ± 8.60	0.93	119 ± 8.36	123 ± 7.44	**< 0.001**
24-hour DBP (mmHg)	68.1 ± 5.53	67.4 ± 5.59	0.12	69.4 ± 6.34	69.8 ± 5.95	0.47
24-hour Heart rate (beats/min)	81.2 ± 8.82	76.5 ± 10.1	**< 0.001**	68.8 ± 8.68	70.2 ± 9.40	0.079
**Anthropometric measurements**						
Weight (kg)	67.7 ± 15.8	68.1 ± 16.1	0.070	64.6 ± 13.0	85.9 ± 16.1	**< 0.001**
Height (cm)	159 ± 6.05	166 ± 6.17	**< 0.001**	169 ± 6.61	179 ± 6.28	**< 0.001**
BMI (kg/m^2^)	26.1 (18.1–39.7)	24.0 (18.3–35.3)	**< 0.001**	22.0 (17.3–30.0)	26.3 (20.2–35.9)	**< 0.001**
WC (cm)	77.9 (61.8–102)	75.7 (63.4–100)	**0.017**	76.2 (64.2–94.0)	88.6 (73.9–110)	**< 0.001**
**Biochemical measurements**						
LDL- C (mmol/L)	2.24 (1.13–3.74)	2.42 (1.25–4.28)	**0.012**	1.88 (0.87–3.62)	2.49 (2.28–4.71)	**< 0.001**
HDL- C (mmol/L)	1.19 ± 0.36	1.35 ± 0.45	**< 0.001**	1.08 ± 0.38	0.96 ± 0.33	**< 0.001**
TC (mmol/L)	3.66 ± 0.88	4.10 ± 1.20	**< 0.001**	3.25 ± 1.00	3.98 ± 1.44	**< 0.001**
Triglycerides (mmol/L)	0.63 (0.34–1.33)	0.76 (0.32–1.84)	**< 0.001**	0.65 (0.28–1.50)	0.86 (0.34–2.22)	**< 0.001**
GGT (U/L)	21.1 (8.75–61.7)	12.2 (5.00-35.5)	**< 0.001**	23.4 (8.30–82.9)	18.5 (6.37–52.7)	**< 0.001**
Glucose (mmol/L)	4.18 ± 0.90	4.24 ± 1.00	0.43	3.63 ± 1.08	4.25 ± 1.16	**< 0.001**
HbA1c (%)	5.47 ± 0.29	5.21 ± 0.27	**< 0.001**	5.40 ± 0.29	5.17 ± 0.28	**< 0.001**
C-reactive protein (mg/l)	1.77 (0.15–14.9)	0.50 (0.08–10.8)	**< 0.001**	0.56 (0.06–5.53)	0.63 (0.07–5.93)	0.30
Cotinine (ng/ml)	1.98 (1-177)	2.22 (1-253)	0.43	8.46 (1-455)	4.61 (1-338)	**0.005**
**Kidney variables**						
s(P)RR (ng/ml)	21.8 (16.6–29.2)	22.9 (17.2–31.2)	**< 0.001**	22.0 (16.9–29.8)	24.5 (19.1–32.2)	**< 0.001**
eGFR (ml/min/1.73m^2^)	120 ± 16.9	115 ± 23.5	**0.001**	132 ± 20.1	123 ± 27.1	**< 0.001**
uACR (mg/mmol)	0.52 (0.16–2.29)	0.55 (0.19–2.79)	0.43	0.49 (0.14–2.29)	0.40 (0.15–1.64)	**0.002**
uA1M (ng/ml)	29.6 (5.04–105)	33.4 (6.88–148)	0.15	50.1 (9.68–209)	39.4 (728 − 143)	**0.003**
**Electrolytes**						
24-hour urinary Na^+^ (mmol/L)	141 ± 66.5	112 ± 64.6	**< 0.001**	147 ± 71.1	121 ± 60.1	**< 0.001**
24-hour urinary K^+^ (mmol/L)	37.8 (11.1–108)	52.7 (13.6–142)	**< 0.001**	32.2 (8.03-93.0)	50.5 (14.7–137)	**< 0.001**
24-hour urinary Na^+^: K^+^ratio	3.40 (1.38–10.1)	1.87 (0.46–5.74)	**< 0.001**	4.16 (1.52–10.9)	2.12 (0.72–6.75)	**< 0.001**
**Lifestyle factors**						
Alcohol n/total (%)	146/292 (50.0%)	160/305 (52.5%)	0.55	169/275 (61.5%)	165/276 (59.8%)	0.68
Smoking n/total (%)	26/296 (8.8%)	50/305 (16.4%)	**0.005**	117/277 (42.2%)	77/277 (27.8%)	**< 0.001**
TEE(kCal/day)	21.2 (15.6–29.8)	21.0 (16.0-31.1)	0.54	22.3 (18.0-28.6)	25.6 (19.7–33.1)	**< 0.001**

Values are presented as arithmetic mean ± standard deviation, geometric mean (5th & 95th percentiles) and number (n) of participants (%)

Bold p-values indicate statistical significance *p* < 0.05

Abbreviations: SES, socio-economic status; BMI, body mass index; WC, waist circumference; TC, total cholesterol; LDL-C, low density lipoprotein-cholesterol; HDL-C, high density lipoprotein-cholesterol; TC, total cholesterol; GGT, gamma-glutamyltransferase; HbA1c, glycosylated haemoglobin; s(P)RR, soluble (pro)renin receptor; eGFR, estimated glomerular filtration rate; uACR, urine albumin-creatinine ratio; uA1M, urine alpha 1-microglobulin; SBP, systolic blood pressure; DBP, diastolic blood pressure; 24-hour urinary Na^+^, sodium; 24-hour urinary K^+^, potassium; 24-hour urinary Na^+^: K^+^ ratio, sodium: potassium ratio; TEE, total energy expenditure; N, number of participants

**Fig. 1:**
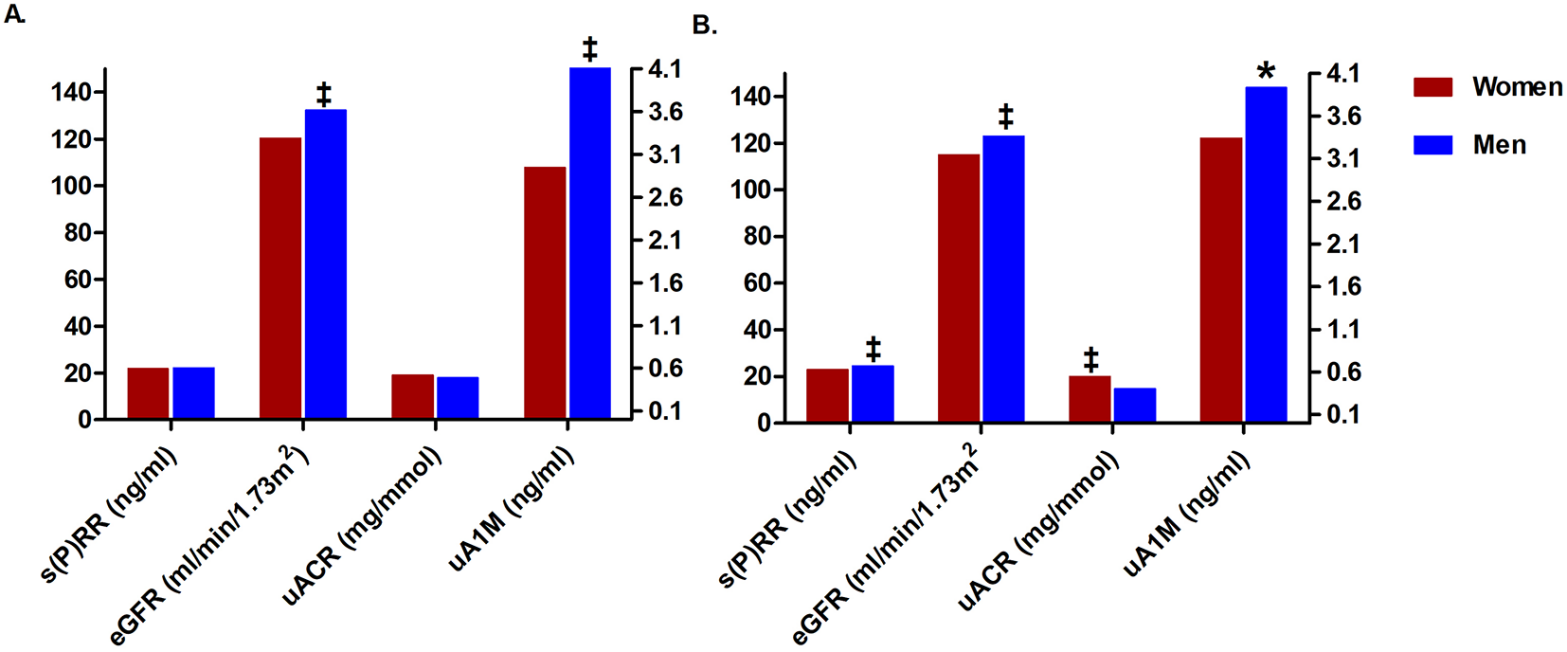
Sex comparisons between kidney function markers and soluble (pro)renin receptor in (**A**) Black and (**B**) White participants. *Abbreviations*: s(P)RR, soluble (pro)renin receptor; uA1M, urine alpha 1-microglobulin; uACR, urine albumin-creatinine ratio; eGFR, estimated glomerular filtration rate ‡ *p* < 0.001; * *p* < 0.05

Figure 1 displays the sex and ethnic differences in the dependent and outcome variables, namely eGFR, uACR, uA1M and s(P)RR. s(P)RR, uA1M and eGFR were higher in White men than in White women, whereas uACR was higher in White women than in White men (all *p* ≤ 0.043). Black men had higher eGFR and uA1M than Black women (both *p* ≤ 0.001).

Table 2 presents the Pearson and partial correlations of kidney function markers and s(P)RR in groups stratified by ethnicity and sex. eGFR negatively correlated with s(P)RR in both bi-ethnic men and women (all *p* ≤ 0.013). Negative correlations remained only in Black women after adjusting for age, WC and triglycerides (*p* < 0.001). There was a positive correlation between uA1M and s(P)RR in White women after adjusting for age, WC and triglycerides (*p* = 0.045). No correlation was observed between uACR and s(P)RR in any of the groups.

**Table 2:** Correlations between soluble (pro)renin receptor and markers of kidney function stratified by sex and ethnicity

	s(*P*)RR (ng/ml)				
	Women *N* = 601		Men *N* = 555		
	Black *N* = 296	White *N* = 305	Black *N* = 278		White *N* = 277
Dependent variable (s)					
eGFR (ml/min/1.73m^2^)	***r*** **= -0.299** ***p*** **< 0.001**	***r*** **= -0.142** ***p*** **= 0.013**	***r*** **= -0.209** ***p*** **< 0.001**		***r*** **= -0.239** ***p*** **< 0.001**
uACR (mg/mmol)	*r*= -0.024 *p* = 0.68	*r*= -0.030 *p* = 0.6	*r*= -0.044 *p* = 0.46		*r*= -0.041 *p* = 0.49
uA1M (ng/ml)	*r* = 0.091 *p* = 0.12	*r*= 0.071 *p* = 0.21	*r*= -0.074 *p* = 0.21		*r* = 0.052 *p* = 0.39
Correlations after applying adjustments for age, WC and triglycerides					
eGFR (ml/min/1.73m^2^)	***r*** **= -0.213** ***p*** **< 0.001**	*r*= -0.055 *p* = 0.34	*r*= -0.109 *p* = 0.072		*r*= -0.078 *p* = 0.20
uACR (mg/mmol)	*r*= -0.038 *p* = 0.52	*r*= -0.008 *p* = 0.89	*r*= -0.032 *p* = 0.58		*r* = 0.013 *p* = 0.82
uA1M(ng/ml)	*r*= 0.103 *p* = 0.09	***r*** **= 0.115** ***p*** **= 0.045**	*r*= -0.076 *p* = 0.20		*r* = 0.117 *p* = 0.054

Bold r-values and p-values indicate statistical significance *p* < 0.05

Abbreviations: s(P)RR, soluble (pro)renin receptor; eGFR, estimated glomerular filtration rate; uACR, urine albumin-creatinine ratio; uA1M, urine alpha 1-microglobulin; WC, waist circumference; N, number of participants

Appropriate interaction tests were performed using ANCOVA to test for ethnicity or sex interactions on the association between the dependent (markers of kidney function namely, eGFR, uACR, and A1M) and independent (s(P)RR) variables. No significant interactions existed between ethnicity/sex and eGFR, uACR, uA1M and s(P)RR (all *p* > 0.05).

**Fig. 2:**
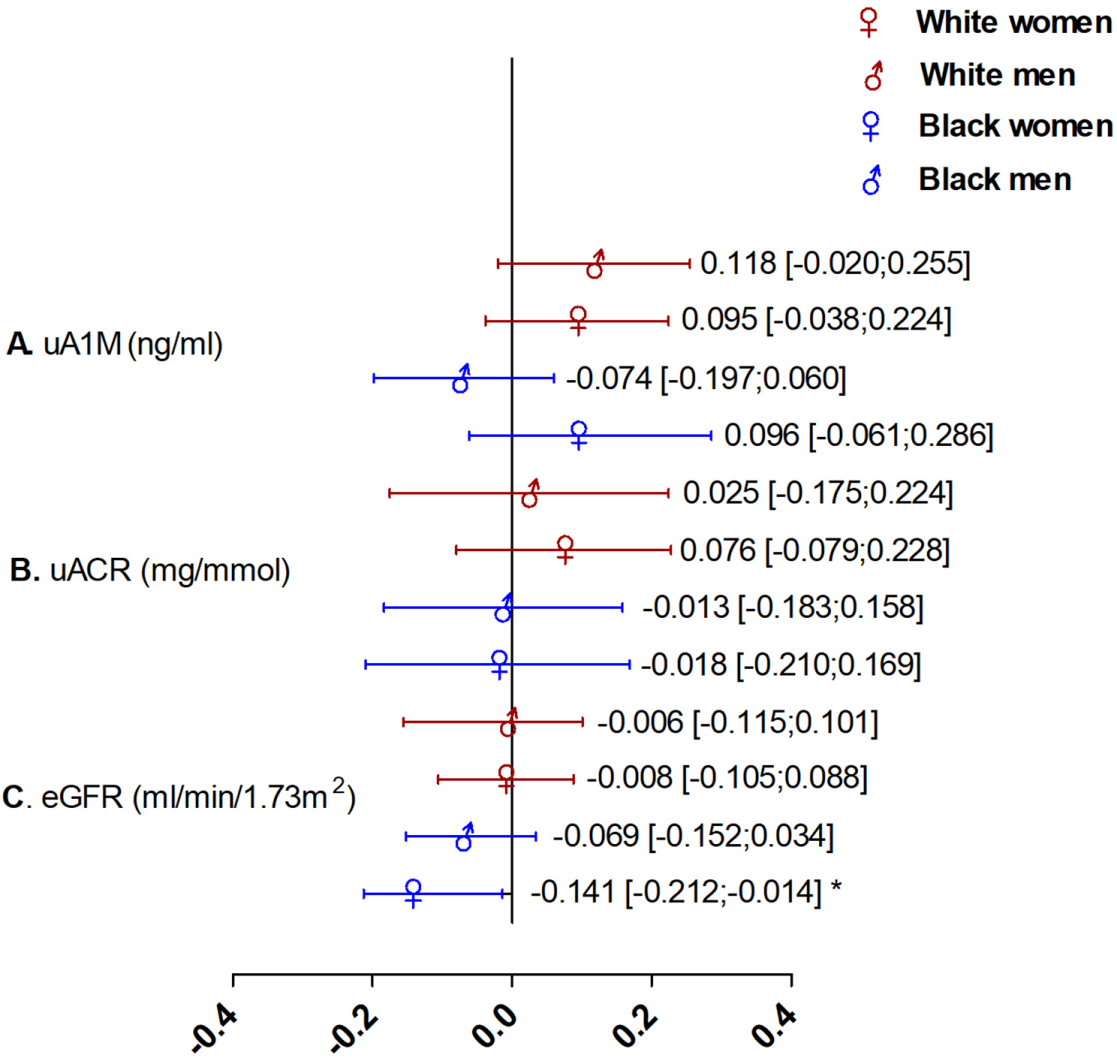
Independent associations between kidney function markers and s(P)RR with the population stratified according to ethnicity and sex. **A** uAIM, **B** uACR and **C** eGFR. Abbreviations: s(P)RR, soluble (pro)renin receptor; uA1M, urine alpha 1-microglobulin; uACR, urine albumin-creatinine ratio; eGFR; estimated glomerular filtration rate. All models were adjusted for age, socio-economic score, 24-hour systolic blood pressure, triglycerides, glucose, cotinine, C-reactive protein, gamma-glutamyltransferase, total energy expenditure and 24-hour urinary sodium: potassium ratio. * *p* < 0.05

Independent associations between markers of kidney function and s(P)RR after adjustments for multiple covariates and confounders are shown in Fig. 2. An independent negative association was observed between s(P)RR and eGFR in Black women only (Adj.R^2^ = 0.309; Std. β=-0.141; *p* = 0.026). The multiple regression analysis results in the White and Black groups are presented in Supplementary Table 3.

## Discussion

In this study we compared s(P)RR levels between participants according to ethnicity and sex and investigated associations of s(P)RR with markers of kidney function (eGFR, uACR and uA1M) in young Black and White men and women. The main findings of the study are the higher s(P)RR levels in both White men and women than in Black men and women, respectively, and higher s(P)RR levels in White men than in White women. In addition, an independent negative association was obtained between eGFR and s(P)RR only in Black women, while uA1M associated positively with s(P)RR only in White participants.

### Ethnic and sex specific differences

Our findings on ethnic differences pertaining to s(P)RR levels are consistent with previous studies in Black and White populations [20, 21], in which s(P)RR levels were higher in young, White adults than in their Black counterparts. Nguyen et al. [20], conducted a study on a very small sample of young, healthy White (*N* = 10) and Black (*N* = 9) subjects (18–35 years) and showed that healthy White men had higher levels of s(P)RR than in their Black counterparts. Similarly, Gafane-Matemane et al. [21], compared s(P)RR levels between Black and White adults in a sub-study of the African-PREDICT study and found higher levels of s(P)RR in young, healthy White adults than in their Black counterparts. Furthermore, our findings revealed that Black individuals had higher eGFR, uACR and uA1M levels compared to White individuals. These findings align with recent studies in young populations [46–48] and with previous population-based studies indicating that conditions that predispose individuals to impaired kidney function are more common in Black than in White populations [49].

Although studies by Nguyen et al. [20], and Morimoto et al. [17], reported that plasma s(P)RR levels did not differ significantly between men and women, our study that found White men had higher s(P)RR levels than their women counterparts; however, there was no difference in s(P)RR levels in the Black group. Our results are consistent with a study by Visniauskas et al. [22], that included *N* = 173 controls and *N* = 96 type 2 diabetes mellitus (T2DM) participants (42% men and 58% women) and found higher levels of plasma s(P)RR in participants with T2DM compared to controls and by a little, but statistically significant amount in men compared to women. Higher urine s(P)RR levels were found in men than women of the control group making it comparable to our health population [22]. Additionally, we found higher eGFR and uA1M levels in White men than in White women and in Black men than in Black women. Our results of uACR and uA1M levels being higher in men are consistent with studies by Hong et al. [30], Zhang et al. [50], and Craig et al. [48]. Taken altogether, our study and previous observations, although under different health conditions confirm that eGFR, uACR and uA1M are sex-dependent. According to Andersson et al. [51], in a study that included 29 healthy individuals, the difference may, in part, be attributed to the male body height and weight, which influences kidney size.

### Associations between markers of kidney function and s(P)RR levels

To the best of our knowledge, this is the first study to demonstrate the ethnic- and sex- specific relationship between uA1M and serum s(P)RR in healthy, young adults. Although the exact mechanism influencing this association was not determined in the present study, it could be explained in the following two ways. Firstly, s(P)RR contributes to inflammation and fibrosis in the interstitial region of the kidney via nicotinamide adenine dinucleotide phosphate (NADPH) oxidase activity, which results in increased oxidative stress [52]. Oxidative stress is known to increase A1M levels and during inflammation it is reported that circulating A1M concentrations are markedly elevated [53]. Secondly, s(P)RR enhances the expression of cytokines such as interleukin (IL)-6 via various signalling pathways [54, 55] that promote gradual inflammatory processes. Inflammation damages kidney cells and impairs the kidney’s ability to reabsorb A1M, leading to an increased urinary excretion of A1M. In addition, s(P)RR stimulates the synthesis of A1M by promoting the expression of IL-6 [53]. As previously reported [21, 45], Ang II levels are two-fold higher in White participants compared to Black participants. A study by Morimoto et al. [17], suggested that elevated serum s(P)RR levels may indicate increased expression of (P)RR in the kidneys, which could lead to higher local production of Ang II by activating prorenin. We therefore speculate this association may be particularly driven and specific to the White group due to Ang II- dependent effects.

Despite the observed levels of uA1M being higher in White men and Black men, they were within the normal range (< 30 mg/L) [55]. This is consistent with prior studies by Takagi et al. [56], and Itoh,1981 [34] who reported quite low concentrations of A1M in healthy individuals. Older individuals, and patients with kidney tubular disorders and CKD had increased amounts of A1M in serum and urine [32, 57–59]. Kawai et al. [60], suggested that latent kidney dysfunction, such as low glomerular filtration, may be the cause of a greater uA1M level in older individuals; therefore, uA1M seems to predict kidney function decrease and CKD development [59, 61].

Although we found no significant associations between some markers of kidney function (uACR) and s(P)RR in any of our study groups, a negative association between eGFR and s(P)RR was observed, which is consistent with previous findings [17, 18, 24]. Serum creatinine, urea nitrogen, uric acid (UA) levels and their urinary protein/Cr ratios were found to be positively associated with s(P)RR, and eGFR was negatively associated with s(P)RR in 374 patients with CKD [18]. In addition, serum s(P)RR was negatively associated with eGFR independent of other factors such as total kidney volume [24]. The relationship between basal s(P)RR levels and renal dysfunction showed that s(P)RR levels were inversely associated with eGFR, suggesting that s(P)RR may be involved in kidney injury and promote the progression of CKD [18]. In these clinical studies, a negative association of s(P)RR with eGFR signals a contribution to a decline in kidney function. On the other hand, the reason for this association in our current study population with normal kidney function is unclear. However, elevated serum s(P)RR levels may result from decreased clearance of s(P)RR by the kidneys [17], which may be applicable to young healthy populations as in the current study where White men had higher eGFR compared to white women, with corresponding higher levels of s(P)RR.

There are several reasonable explanations for the contrasting results observed in our study and these include the following: firstly, the eGFR of most participants (*N* = 1156) included in this study was > 90 ml/min per 1.73m^2^ indicating that the mean kidney function is within the normal range [62, 63]. Secondly, participants were young (20–30 years old), and it is well known that kidney function of people > 60 years is lower than that of younger people [59, 64]. Thirdly, although uACR levels were higher in Black men than in White men, the levels were within the normal range (≤ 1.75 mg/mmol) [65]. And lastly, our study population consisted of a population with no known diseases and no history of taking any chronic medication. It has been established that s(P)RR levels are elevated in CKD and CVD, with a negative association with eGFR and a positive association with proteinuria. Although the current study population did not include patients with confirmed kidney damage or disease, this study makes a significant contribution to the field of knowledge on s(P)RR as novel biomarker for CKD and CVD as well as target for therapeutic interventions to aid in primary prevention at younger ages and treatment of target organ damage.

Our study should be considered within the context of its strengths and limitations. The study was carried out under highly controlled conditions in a well-equipped research facility, and we used a sizeable study group to ensure sufficient power for statistical analyses and a comparable sex distribution, which allowed us to evaluate ethnic and sex differences in s(P)RR levels and kidney function. In addition, the young population was healthy with no known acute and/or chronic diseases and without any history of kidney and cardiovascular diseases. The limitations of our study are that the research population was recruited from Potchefstroom and surrounding areas of the North West Province, with a strict age group and therefore is not representative of the entire population of young South African adults. As such the results cannot be generalised for all South Africans. The cross-sectional design of the study means the associations cannot be used to infer any causality and only suggest possible mechanisms underlying the associations. We acknowledge that we did not account for the well-known low RAAS activity in Black individuals which may among others be due to high Na^+^ and low K^+^ due to high dietary salt intake which could influence the results. Lastly, our results were consistent even after multiple adjustments for known confounders, however residual confounding cannot be excluded.

## Conclusions

In conclusion, our study confirmed ethnic and sex differences in s(P)RR levels, which were higher in White men and women than in their Black counterparts and higher in men than in women but only in the White group. In this group of young adults, a negative association between eGFR and s(P)RR was observed in young Black women only and a positive association was observed between uA1M and s(P)RR in White participants. Our findings highlight the potential role of s(P)RR as a biomarker of kidney function which is influenced by ethnicity and sex. Longitudinal and population wide studies are recommended to determine the validity and predictive value of s(P)RR as a risk marker.

## Electronic supplementary material

Below is the link to the electronic supplementary material.

Supplementary Material 1: Additional file 1: Supplementary Table 1 presents the comparison of study population characteristics between the Black and White groups. s(P)RR was higher in the White group, while eGFR was higher in the Black group (both p<0.001).

Supplementary Material 2: Additional File 2: The Pearson and partial correlations between markers of kidney function and s(P)RR in study population stratified by ethnicity are shown in Additional file 2: Supplementary Table 2. A negative correlation between eGFR and s(P)RR was observed in both Black and White participants (p≤0.001). After adjusting for age, WC and triglycerides, a negative correlation between eGFR and s(P)RR was observed in only Black participants (p=0.001). uA1M associated positively with s(P)RR in White participants after adjusting for age, sex and WC (p=0.002). Furthermore, no significant correlation existed between uACR and s(P)RR in both Black and White participants.

Supplementary Material 3: Additional File 3: In addition, we explored the association between markers of kidney function and s(P)RR in a study population stratified by ethnicity (Additional file 3: Supplementary Table 3). uA1M associated positively with s(P)RR in White participants only (Adj.R-squared=0.063; Std. β=0.115; p=0.018). No association was observed between other markers of kidney function (uACR and eGFR) and s(P)RR in either Black or White participants.

## Data Availability

The dataset analyzed during the current study is available from the corresponding author on reasonable request.

## Abbreviations

ABPM: Ambulatory Blood Pressure Monitoring
African-PREDICT: African Prospective study on the Early Detection and Identification of Cardiovascular disease and Hypertension
A1M: Alpha 1-Microglobulin
uA1M: Urine Alpha 1-Microglobulin
Ang II: Angiotensin II
BMI: Body Mass Index
BP: Blood Pressure
CKD: Chronic Kidney Disease
CKD-EPI: Chronic Kidney Disease Epidemiology Collaboration
CRP: C-Reactive Protein
DBP: Diastolic Blood Pressure
eGFR: Estimated Glomerular Filtration Rate
GGT: Gamma-Glutamyltransferase
HART: Hypertension in Africa Research Team
HbA1c: Glycated Haemoglobin
HDL-C: High Density Lipoprotein-Cholesterol
HIV: Human Immunodeficiency Virus
HREC: Human Research Ethics Committee
IL-6: Interleukin-6
ISAK: International Society for The Advancement of Kinanthropometry
K^+^: Potassium
LDL-C: Low Density Lipoprotein-Cholesterol
Na^+^: Sodium
Na^+^: K^+^: Sodium: Potassium Ratio
NADPH: Nicotinamide Adenine Dinucleotide Phosphate
NF-B: Nuclear Factor-B
PAI-1: Plasminogen Activator Inhibitor-1
(P)RR: (Pro)renin Receptor
RAAS: Renin Angiotensin-Aldosterone System
SAMRC: South African Medical Research Council
SBP: Systolic Blood Pressure
SES: Socio-Economic Status
s(P)RR: Soluble (Pro)renin Receptor
T2DM: Type 2 Diabetes Mellitus
TC: Total Cholesterol
TEE: Total Energy Expenditure
uACR: Urine Albumin-Creatinine Ratio
WC: Waist Circumference
