# Supplementary material for: The relationship between kidney function and the soluble (pro)renin receptor in young adults: the African-PREDICT study
Source: BMC Nephrol. 2025 Apr 3;26:172. doi: 10.1186/s12882-025-04038-x (PMC11966904; doi:10.1186/s12882-025-04038-x)
Supplement: Supplementary file 1 — Supplementary Material 1: Additional file 1: Supplementary Table 1 presents the comparison of study population characteristics between the Black and White groups. s(P)RR was higher in the White group, while eGFR was higher in the Black group (both p<0.001). [file 12882_2025_4038_MOESM1_ESM.docx]

**Supplementary Table 1** The comparison of the characteristics of the study participants stratified by ethnicity

|  | Black | White | p-value |
| --- | --- | --- | --- |
|  | N=574 | N=582 |  |
| **Socio-demographic profile** | | | |
| Age (years) | 24.5 ± 3.17 | 24.6 ± 3.04 | 0.56 |
| Sex female n/total (%) | 296/574 (51.6%) | 305/582 (52.4%) | 0.78 |
| SES class |  |  |  |
| Low n/total (%)  Middle n/total (%)  High n/total (%) | 337/573 (58.8%) | 114/582 (39.0%) | **<0.001** |
|  | 157/573(27.4%) | 182/582 (31.3%) |  |
|  | 79/573 (13.8%) | 286/582 (49.1%) |  |
| **Blood pressure measurements** | | | |
| 24-hour SBP (mmHg) | 115 ± 9.10 | 118 ± 9.83 | **0.001** |
| 24-hour DBP (mmHg) | 68.7 ± 5.96 | 68.5 ± 5.88 | 0.58 |
| 24-hour Heart rate (beats/min) | 75.3 ± 10.7 | 73.5 ± 10.3 | **0.005** |
| **Anthropometric measurements** | | | |
| Weight (kg) | 66.2 ± 14.6 | 76.6 ± 18.4 | **<0.001** |
| Height (cm) | 164 ± 8.35 | 172 ± 8.84 | **<0.001** |
| Body mass index (kg/m^2^) | 24.0 (17.6-35.5) | 25.1 (18.8-35.6) | **<0.001** |
| Waist circumference (cm) | 77.1 (62.9-97.0) | 81.6 (65.1-107) | **<0.001** |
| **Biochemical measurements** | | | |
| LDL- C (mmol/L) | 2.06 (0.98-3.70) | 2.45 (1.20-4.42) | **<0.001** |
| HDL- C(mmol/L) | 1.13 ± 0.37 | 1.17 ± 0.45 | 0.18 |
| Total cholesterol (mmol/L) | 3.46 ± 0.96 | 4.04 ± 1.32 | **<0.001** |
| Triglycerides (mmol/L) | 0.64 (0.31-1.36) | 0.80 (0.33-2.08) | **<0.001** |
| Gamma-glutamyltransferase (U/L) | 22.2 (8.47-66.4) | 14.9 (5.40-46.9) | **<0.001** |
| Glucose (mmol/L) | 3.91 ± 1.03 | 4.25 ± 1.08 | **<0.001** |
| Glycosylated haemoglobin (%) | 5.44 ± 0.29 | 5.19 ± 0.28 | **<0.001** |
| C-reactive protein (mg/l) | 1.02 (0.08-11.6) | 0.77 (0.08-8.08) | **0.002** |
| Cotinine (ng/ml) | 4.00 (1-333) | 3.15 (1-305) | 0.069 |
| **Kidney variables** | | | |
| s(P)RR (ng/ml) | 21.9(16.8 29.3) | 23.7 (17.6-31.6) | **<0.001** |
| eGFR (ml/min/1.73m^2^) | 126 ± 19.5 | 118 ± 25.6 | **<0.001** |
| uACR (mg/mmol) | 0.51 (0.15-2.23) | 0.48 (0.16-2.33) | 0.16 |
| uA1M (ng/ml) | 38.2 (6.72-184) | 36.1 (7.09-144) | 0.36 |
| **Electrolytes** | | | |
| 24-hour urinary Na^+^ (mmol/L) | 144 ± 68.8 | 116 ± 62.6 | **<0.001** |
| 24-hour urinary K^+^ (mmol/L) | 35.0 (9.53-100) | 51.6 (14.1-142) | **<0.001** |
| 24-hour urinary Na^+^: K^+^ ratio | 3.68 (1.43-10.5) | 1.97 (0.50-6.16) | **<0.001** |
| **Lifestyle factors** | | | |
| Alcohol n/total (%) | 315/567 (55.6%) | 325/581 (55.9%) | 0.89 |
| Smoking n/total (%) | 143/573 (25.0%) | 127/582 (21.8%) | 0.20 |
| Total energy expenditure (kCal/day) | 21.7 (16.7-28.8) | 23.0 (16.8-32.8) | **<0.001** |
| Values are interpreted as arithmetic mean ± standard deviation, geometric mean (5th & 95th percentiles) and number (n) of participants (%)  Bold p-values indicate statistical significance p≤0.05  Abbreviations: SES, socio-economic status; LDL-C, low density lipoprotein-cholesterol; HDL-C, high density lipoprotein-cholesterol; s(P)RR, soluble (pro)renin receptor; eGFR, estimated glomerular filtration rate; uACR, urine albumin-creatinine ratio; uA1M, urine alpha 1-microglobulin; SBP, systolic blood pressure; DBP, diastolic blood pressure; 24-hour urinary Na+, Sodium; 24-hour urinary K^+^, Potassium; 24-hour urinary Na^+^: K^+^ ratio, sodium: potassium ratio; N, number of participants | | | |
