# Supplementary material for: The relationship between kidney function and the soluble (pro)renin receptor in young adults: the African-PREDICT study
Source: BMC Nephrol. 2025 Apr 3;26:172. doi: 10.1186/s12882-025-04038-x (PMC11966904; doi:10.1186/s12882-025-04038-x)
Supplement: Supplementary file 2 — Supplementary Material 2: Additional File 2: The Pearson and partial correlations between markers of kidney function and s(P)RR in study population stratified by ethnicity are shown in Additional file 2: Supplementary Table 2. A negative correlation between eGFR and s(P)RR was observed in both Black and White participants (p≤0.001). After adjusting for age, WC and triglycerides, a negative correlation between eGFR and s(P)RR was observed in only Black participants (p=0.001). uA1M associated positively with s(P)RR in White participants after adjusting for age, sex and WC (p=0.002). Furthermore, no significant correlation existed between uACR and s(P)RR in both Black and White participants. [file 12882_2025_4038_MOESM2_ESM.docx]

**Supplementary Table 2** Correlations between markers of kidney function and soluble (pro)renin receptor stratified by ethnicity

| s(P)RR (ng/ml) | | |
| --- | --- | --- |
|  | Black  N=574 | White  N= 582 |
| Dependent variable(s) |  |  |
| eGFR (ml/min/1.73m^2^) | **r= -0.230**  **p<0.001** | **r= -0.154**  p= **0.001** |
| uACR (mg/mmol) | r= -0.032  p= 0.44 | r= -0.069  p= 0.09 |
| uA1M (ng/ml) | r= 0.022  p= 0.59 | r= 0.077  p=0.064 |
| Correlation after adjustment for age, WC and triglycerides | | |
| eGFR (ml/min/1.73m^2^) | **r= -0.135**  **p=** **0.001** | r= -0.051  p= 0.218 |
| uACR (mg/mmol) | r= -0.025  p= 0.54 | r= 0.009  p= 0.82 |
| uA1M (ng/ml) | r= 0.028  p= 0.50 | **r= 0.127**  **p=0.002** |
| Bold r-values and p-values indicate statistical significance p≤0.05  Abbreviations: s(P)RR, soluble (pro)renin receptor; eGFR, estimated glomerular filtration rate; uACR, urine albumin-creatinine ratio; uA1M, urine alpha 1-microglobulin; N, number of participants | | |
