# Supplementary material for: The relationship between kidney function and the soluble (pro)renin receptor in young adults: the African-PREDICT study
Source: BMC Nephrol. 2025 Apr 3;26:172. doi: 10.1186/s12882-025-04038-x (PMC11966904; doi:10.1186/s12882-025-04038-x)
Supplement: Supplementary file 3 — Supplementary Material 3: Additional File 3: In addition, we explored the association between markers of kidney function and s(P)RR in a study population stratified by ethnicity (Additional file 3: Supplementary Table 3). uA1M associated positively with s(P)RR in White participants only (Adj.R-squared=0.063; Std. β=0.115; p=0.018). No association was observed between other markers of kidney function (uACR and eGFR) and s(P)RR in either Black or White participants. [file 12882_2025_4038_MOESM3_ESM.docx]

**Supplementary Table 3** Multiple linear regression analysis between kidney function markers and soluble (pro)renin receptor according to ethnicity

|  | Black  N=574 | | | White  N=582 | | | |
| --- | --- | --- | --- | --- | --- | --- | --- |
|  | Adj. R^2^ | Std. β (±95% CI) | p-value | | Adj. R^2^ | Std. β (±95% CI) | p-value |
| eGFR (ml/min/1.73m^2^) | | | | | | | |
| **s(P)RR (ng/ml)** | 0.446 | -0.069 (-0.152 0.034) | 0.21 | | 0.658 | -0.006 (-0.115 0.101) | 0.89 |
|  | uACR (mg/mmol) | | | | | | |
| **s(P)RR (ng/ml)** | 0.023 | 0.010 (-0.135 0.113) | 0.86 | | 0.062 | 0.053 (-0.067 0.173) | 0.38 |
|  | uA1M (ng/ml) | | | | | | |
| **s(P)RR (ng/ml)** | 0.061 | 0.024 (-0.081 0.133) | 0.63 | | 0.063 | 0.115 (0.019 0.205) | **0.018** |
| Bold p-values indicate statistical significance p≤0.05  All models were adjusted for Age, socio-economic score, 24-hour systolic AMBP, triglycerides, glucose, cotinine, C-reactive protein, gamma-glutamyltransferase, total energy expenditure and 24-hour urinary sodium: potassium ratio  Abbreviations: s(P)RR, soluble (pro)renin receptor; eGFR, estimated glomerular filtration rate; uACR, urine albumin-creatinine ratio; uA1M, urine alpha 1-microglobulin; N, number of participants | | | | | | | |
